# Cardiovascular risk assessed by the conicity index in Brazilian adults: findings from the national health survey

**DOI:** 10.3389/fnut.2026.1688180

**Published:** 2026-01-30

**Authors:** Yasmin Franco Rodrigues Silva, Camila Bruneli do Prado, Virgínia Maria Muniz, Fabiano Kenji Haraguchi, Luciane Bresciani Salaroli

**Affiliations:** 1 Graduate Program in Nutrition and Health, Federal University of Espírito Santo, Vitória, Brazil; 2 Graduate Program in Public Health, Federal University of Espírito Santo, Vitória, Brazil

**Keywords:** abdominal obesity, anthropometry, body composition, cardiovascular risk, central obesity, Framingham risk score

## Abstract

**Background:**

Anthropometric indices such as the Conicity Index (C-Index) are emerging as accessible tools to assess cardiovascular risk associated with central adiposity, a key determinant of cardiovascular disease burden in low and middle-income countries.

**Aim:**

To estimate the prevalence of cardiovascular risk using the CI and examine its association with sociodemographic and lifestyle factors in a nationally representative sample of Brazilian adults.

**Methods:**

In this cross-sectional study using data from the 2013 Brazilian National Health Survey (*n* = 42,693; ages 30–74), cardiovascular risk was defined based on sex-specific CI cut-off points.

**Results:**

The overall prevalence of elevated cardiovascular risk was 39.6%, with a significantly higher rate among women (64.6%) compared to men (35.4%). Increased age, lower education, lower income, poor self-rated health, and physical inactivity were independently associated with greater cardiovascular risk, with regional and sex-based differences observed.

**Conclusion:**

The CI proved to be a practical, non-invasive measure strongly associated with key social determinants of health and behavioral risk factors. These findings support its integration into public health monitoring and preventive strategies to identify at-risk groups, particularly in resource-constrained settings.

## 1 Introduction

Cardiovascular diseases (CVD) remain the leading cause of mortality worldwide and a major contributor to disability (1). The number of CVD cases nearly doubled, increasing from 271 million in 1990 to 523 million in 2019, while deaths rose from 12.1 million to 18.6 million during the same period (2). This increase was observed globally, with a greater impact in low- and middle-income countries (3, 4). Assessing cardiovascular risk factors is essential for the proper prevention and management of these conditions (5). In this context, the conicity index (C-Index), a measure of central adiposity, has been studied as a potential indicator of cardiovascular risk (6, 7).

This index is based on the geometric concept of a double cone, suggesting that the higher the C-Index, the greater the concentration of fat in the abdominal region, which is directly associated with cardiovascular disease risk (8–10). Visceral fat, which accumulates around abdominal organs, exerts metabolic effects that directly contribute to increased blood pressure (11, 12), dyslipidemia (13) and insulin resistance (14) and has been identified as an important risk factor for morbidity and mortality (15). Thus, the C-Index stands out as a promising alternative, combining waist circumference, weight, and height to provide a more accurate assessment of visceral fat and cardiovascular risk (16–18).

When compared to other anthropometric measures, such as waist-to-hip ratio and waist-to-height ratio, some studies suggest that the C-Index may be more effective in predicting cardiovascular risk, especially in both men and women over a 10-year period (19, 20). Waist circumference, widely used to assess abdominal obesity, has been recommended as a public health screening tool, particularly in populations predisposed to heart disease (21, 22). However, isolated measures may be insufficient to capture the complexity of body fat distribution (21, 22). Furthermore, socioeconomic factors and lifestyle habits play a fundamental role in determining cardiovascular risk (23–26). Individuals with lower socioeconomic status tend to exhibit a higher prevalence of risk factors such as physical inactivity, inadequate diets, and limited access to healthcare services, which may contribute to increased central adiposity and, consequently, a higher C-Index (27–29).

Although the C-Index is a potential predictor of cardiovascular risk, its application remains underexplored in Brazil (30). Using the C-Index in the Brazilian population is an alternative worth considering, as this tool offers a simple, low-cost, and non-invasive way to identify individuals at higher risk of CVD related to abdominal obesity (31, 32). Therefore, this study aims to estimate the prevalence of cardiovascular risk in the Brazilian population using the C-Index and to investigate its association with sociodemographic variables and lifestyle factors.

## 2 Materials and methods

### 2.1 Study design and sample

This cross-sectional study used secondary data from the 2013 National Health Survey (PNS), a nationally representative household health survey conducted by the Ministry of Health in partnership with the Brazilian Institute of Geography and Statistics (IBGE). The PNS is designed to collect information on various health aspects of the Brazilian population living in private permanent households. The survey was structured in three parts, covering questions related to the household, its residents, and, individually, one adult randomly selected from each household, focusing on topics such as work, social support, lifestyle habits, and chronic diseases (33, 34). For this study, data from the randomly selected adult resident were used, as anthropometric measurements and blood pressure were collected only from this individual. At the end of data collection, 81,167 households were visited, of which 69,994 were occupied, resulting in 64,348 household interviews and 60,202 individual interviews with the selected residents (35).

### 2.2 Inclusion and exclusion criteria

Individuals of both sexes aged 30 to 74 years were included, since the cutoff point for the C-Index was established for this age range based on the Framingham score (36). All individuals with missing data on waist circumference, weight, and height necessary for the calculation of the C-Index were excluded, as well as pregnant women.

### 2.3 Anthropometry measurements

Anthropometric measurements were performed by trained professionals using standardized equipment. Body weight was obtained with participants barefoot, standing upright, and wearing minimal clothing. Height was measured with participants barefoot, standing with legs and feet parallel, weight evenly distributed between both feet, arms relaxed at the sides, and palms facing inward. Waist circumference was measured with the participant standing, arms flexed and crossed in front of the chest, feet apart, using a non-elastic measuring tape positioned at the midpoint between the lower edge of the costal arch and the iliac crest (34).

### 2.4 Variables

The C-Index was calculated using weight, height, and waist circumference measurements through the following mathematical equation (9):


$$\text{Conicity Index}=\frac{\text{Waist circumference}\;(m)}{0.109\sqrt{\frac{\text{Body weight}\;(\mathrm{kg})}{\text{Height}\;(m)}}}$$

The following sociodemographic characteristics were considered: sex (male; female); age group (30–39; 40–49; 50–59; 60–69; 70+); race and ethnicity (White; Black; Brown; East-Asian; Indigenous); highest level of education previously completed (no education/incomplete elementary school; complete elementary school; complete high school; complete higher education/postgraduate degree); marital status (married; divorced/separated; single; widowed); whether you have a paid occupation (yes; no); income range (≤ 1 minimum wage; > 1 and ≤ 2 minimum wages; > 2 and < 5 minimum wages; ≥ 5 minimum wages); region of Brazil where you reside (North; Northeast; Midwest; Southeast; South).

The following variables related to lifestyle habits were considered: Self-rated health (Very good/Good; fair; poor/very poor); Smoking habit (Smoker; non-smoker); Physical activity practice within the recommended range, with the recommendation being 150 min of moderate-intensity activity or at least 75 min of vigorous-intensity physical activity per week (yes; no) (35, 36); Excessive alcohol consumption defined as the intake of five or more alcoholic drinks for men and four or more drinks for women, on a single occasion, in the last 30 days (yes; no) (37, 38).

### 2.5 Statistical analysis

The prevalence of cardiovascular risk was calculated based on C-Index cut-off points previously determined from the Framingham score, set at 1.344 for males and 1.285 for females (36). Prevalence estimates were calculated using survey weights; absolute numbers correspond to unweighted sample counts.

Descriptive analysis was performed separately by sex, with participants classified according to the presence or absence of cardiovascular risk based on the C-Index cut-off. Variables were presented as proportions with 95% confidence intervals (95% CI). Associations between cardiovascular risk and categorical variables were tested using the chi-square test. Variables with *p*-values ≤ 0.05 in bivariate analysis were included in a binary logistic regression model, from which adjusted odds ratios (OR) and their respective 95% CIs were estimated. Results with p-values ≤ 0.05 were considered statistically significant.

The analyses accounted for the complex sampling design of the National Health Survey (Pesquisa Nacional de Saúde—PNS), incorporating sampling weights using the survey package. The adjusted analyses were performed using survey-weighted logistic regression models. The models were adjusted for region, age, race and ethnicity, marital status, education, employment status, income, self-rated health status, and physical activity level. These covariates were selected based on their theoretical relevance and previous evidence as potential confounders in the relationship between sociodemographic characteristics, health behaviors, and obesity.

All analyses were performed in R software (version 4.0.3) for Windows, adopting a significance level of 5%.

## 3 Results

A total of 64,308 individuals were selected for the individual interview; of these, 4,106 were not interviewed due to refusal or difficulty locating them for data collection. Based on the predefined inclusion and exclusion criteria, 800 pregnant women and 16,709 individuals outside the age range of 30–74 years were excluded. There were no individuals with missing data on weight, height, or waist circumference. Therefore, the final sample used in this study comprised 42,693 participants, including 24,117 women and 18,576 men.

The overall prevalence of elevated cardiovascular risk according to the C-Index, in the Brazilian population was 39.6% (95% CI: 38.7–40.5). When stratified by sex, the weighted prevalence was 35.4% (95% CI: 34.0–36.7) in men and 64.6% (95% CI: 63.3–66.0) in women. The highest prevalence was observed in the state of Rio Grande do Norte (51.55%; Table 1).

**Table 1:** Prevalence of cardiovascular risk in the states of Brazil, according to the conicity index, data from the national health survey (2013).

State	%	CI
Rio Grande do Norte	51.56	(47.87–55.25)
Ceará	47.99	(44.53–51.46)
Piauí	47.30	(43.79–50.81)
Sergipe	46.33	(42.88–49.77)
Alagoas	43.55	(40.34–46.76)
São Paulo	43.55	(40.98–46.12)
Santa Catarina	43.41	(39.25–47.57)
Roraima	42.81	(39.01–46.62)
Rio de Janeiro	39.83	(37.19–42.48)
Rio Grande do Sul	39.21	(35.85–42.58)
Mato Grosso do Sul	38.34	(34.96–41.71)
Paraíba	37.57	(33.30–41.83)
Mato Grosso	37.29	(33.71–40.86)
Acre	37.02	(33.67–40.36)
Bahia	36.86	(31.96–41.76)
Pará	36.73	(32.28–41.19)
Goiás	35.99	(32.89–39.08)
Paraná	35.70	(32.04–39.37)
Minas Gerais	35.66	(32.64–38.67)
Pernambuco	35.48	(32.34–38.63)
Distrito Federal	35.10	(32.03–38.16)
Rondônia	35.06	(32.00–38.13)
Tocantins	34.81	(29.18–40.45)
Maranhão	34.68	(30.07–39.29)
Amazonas	33.12	(30.42–35.83)
Espírito Santo	31.84	(27.87–35.81)
Amapá	29.70	(26.03–33.37)

CI, Confidence interval.

Among individuals with cardiovascular risk related to central adiposity, both men and women showed similar characteristics: most resided in the Southeast region, were between 50 and 59 years old, self-identified as White, were married, had low education levels (no schooling or incomplete elementary school), had paid employment, reported a per capita income of less than one minimum wage, rated their health as very good/good, did not report excessive alcohol consumption, were non-smokers, and were physically inactive. There were statistically significant differences between elevated cardiovascular risk according to the C-Index and most qualitative variables, except for alcohol consumption and smoking among men (Tables 2, 3).

**Table 2:** Comparison between sociodemographic variables and lifestyle habits according to the C-index in the Brazilian male population: National Health Survey, Brazil, 2013.

Variable	<1.344		≥1.344		*p*-value
	%	95% CI	%	95% CI	
Regions					**0.0023**
Midwest	7.67	(7.19–8.15)	6.92	(6.14–7.70)	
Northeast	26.61	(25.38–27.84)	23.33	(21.34–25.31)	
North	7.69	(7.17–8.20)	6.57	(5.76–7.39)	
Southeast	43.64	(41.98–45.30)	45.83	(43.33–48.33)	
South	14.39	(13.46–15.32)	17.35	(15.39–19.32)	
Age range					**<0.0001**
30–39	39.75	(38.27–41.22)	13.46	(12.01–14.90)	
40–49	28.11	(26.86–29.37)	19.94	(18.07–21.81)	
50–59	20.11	(18.80–21.43)	33.03	(30.61–35.46)	
60–69	9.23	(8.40–10.05)	24.79	(22.68–26.91)	
70–74	2.8	(2.28–3.31)	8.77	(7.56–9.99)	
Race and ethnicity					**<0.0001**
Yellow	0.83	(0.60–1.06)	0.63	(0.31–0.94)	
White	45.11	(43.60–46.63)	55.17	(52.82–57.51)	
Indigenous	0.35	(0.23–0.46)	0.25	(0.08–0.42)	
Brown	43.11	(41.70–44.53)	36.46	(34.21–38.71)	
Black	10.6	(9.72–11.47)	7.49	(6.21–8.78)	
Marital status					**<0.0001**
Married	55.71	(54.07–57.35)	65.72	(63.58–67.86)	
Divorced/Separated/Widowed	8.68	(7.83–9.53)	12.11	(10.70–67.86)	
Single	35.61	(34.10–37.13)	22.17	(20.34–24.00)	
Education					**<0.0001**
No education/incomplete elementary school	44	(42.41–45.58)	49.17	(46.58–51.76)	
Complete elementary education /incomplete high school	14.23	(13.21–15.24)	12.15	(10.46–13.83)	
Complete high school / Incomplete higher education	28.99	(27.60–30.37)	24.42	(22.42–26.41)	
Complete higher education/postgraduate degree	12.79	(11.66–13.92)	14.26	(12.37–16.15)	
Paid occupation					**<0.0001**
No	21.99	(20.64–23.35)	36.27	(76.65–79.36)	
Yes	78.01	(33.88–38.67)	63.73	(61.33–66.12)	
Income					**<0.0001**
Less than 1 Minimum Wage	47.44	(45.81–49.06)	41.41	(38.97–43.85)	
Between 1 and 2 Minimum Wages	29.79	(28.35–31.23)	30.3	(28.05–32.54)	
Between 2 and 5 Minimum Wages	16.52	(15.39–17.66)	18.51	(16.73–20.30)	
Greater than 5 Minimum Wages	6.25	(5.46–7.04)	9.78	(8.17–11.40)	
Self-rated health					**<0.0001**
Very good/Good	70.13	(68.71–71.56)	57.11	(54.72–59.50)	
Fair	25.5	(24.13–26.87)	35.23	(32.89–37.58)	
Poor/Very poor	4.37	(3.83–4.91)	7.66	(6.57–8.74)	
Alcohol consumption					0.0583
No	78.92	(77.70–80.15)	80.95	(79.20–82.69)	
Yes	21.08	(19.85–22.30)	19.05	(17.31–20.80)	
Smoker					0.3198
No	79.39	(78.20–80.57)	80.59	(78.58–82.60)	
Yes	20.61	(19.43–21.80)	19.41	(17.40–21.42)	
Physical activity					**<0.0001**
No	90.6	(89.76–91.44)	96.29	(95.44–97.14)	
Yes	9.4	(8.56–10.24)	3.71	(2.86–4.56)	

*N* = 18,576; <1.344 = 13,290; ≥1.344 = 5,286. *Chi-square test; CI%, Confidence interval; C-index cutoff point to assess cardiovascular risk: ≥1.344 (men).Bold values indicate *p*-value < 0.05.

**Table 3:** Comparison between sociodemographic variables and lifestyle habits according to the C-index in the Brazilian female population: national health survey, Brazil, 2013.

Variable	<1.285		≥1.285		*p*-value*
	%	95% CI	%	95% CI	
Regions					**<0.0001**
Midwest	7.78	(7.31–8.26)	6.68	(6.20–7.17)	
Northeast	23.79	(22.59–24.99)	27.87	(26.35–29.39)	
North	7.09	(6.65–7.54)	6.04	(5.54–6.53)	
Southeast	45.18	(43.54–46.82)	46.08	(44.36–47.79)	
South	16.15	(15.09–17.21)	13.34	(12.28–14.39)	
Age range					**<0.0001**
30–39	39.6	(38.14–41.07)	19.35	(18.07–20.62)	
40–49	30.25	(28.92–31.58)	22.82	(21.42–24.23)	
50–59	18.41	(17.20–19.62)	27.72	(26.17–29.27)	
60–69	9.4	(8.55–10.25)	22.34	(21.03–23.65)	
70–74	2.33	(1.86–2.80)	7.77	(6.91–8.64)	
Race and ethnicity					**0.0001**
Yellow	1.35	(0.94–1.76)	0.64	(0.43–0.85)	
White	50.18	(48.59–51.76)	46.91	(45.25–48.58)	
Indigenous	0.52	(0.33–0.70)	0.5	(0.30–0.69)	
Brown	39.23	(37.70–40.77)	42.16	(40.51–43.81)	
Black	8.72	(7.89–9.55)	9.79	(8.80–10.78)	
Marital status					**<0.0001**
Married	50.55	(49.01–52.08)	51.54	(0.52–53.21)	
Divorced/Separated/Widowed	16.78	(15.76–17.81)	23.02	(21.68–24.35)	
Single	32.67	(31.25–34.08)	25.44	(0.25–26.80)	
Education					**<0.0001**
No education/incomplete elementary school	34.55	(33.00–36.10)	53.18	(51.38–54.97)	
Complete elementary education /incomplete high school	12.84	(11.84–13.84)	12.82	(11.72–13.92)	
Complete high school / Incomplete higher education	32.33	(30.98–33.69)	23.99	(22.58–25.40)	
Complete higher education/postgraduate degree	20.27	(18.76–21.78)	10.01	(0.10–11.15)	
Paid occupation					**<0.0001**
No	45.44	(44.04–46.84)	61.34	(0.61–63.04)	
Yes	54.56	(53.16–55.96)	38.66	(0.39–40.36)	
Income					**<0.0001**
Less than 1 Minimum Wage	47.53	(45.76–49.31)	53.77	(51.94–55.60)	
Between 1 and 2 Minimum Wages	27.08	(25.72–28.45)	28.1	(26.60–29.60)	
Between 2 and 5 Minimum Wages	17.84	(16.59–19.09)	14	(12.69–15.32)	
Greater than 5 Minimum Wages	7.54	(6.53–8.56)	4.13	(3.53–4.73)	
Self-rated health					**<0.0001**
Very good/Good	66.28	(64.83–67.74)	49.6	(0.50–51.20)	
Fair	28.45	(27.08–29.83)	39.74	(0.40–41.30)	
Poor/Very poor	5.26	(4.64–5.89)	10.65	(0.11–11.62)	
Alcohol consumption					**0.0087**
No	93.66	(92.92–94.40)	94.9	(0.95–95.57)	
Yes	6.34	(5.60–7.08)	5.1	(0.05–5.76)	
Smoker					**0.0023**
No	87.94	(86.99–88.90)	85.67	(0.86–86.75)	
Yes	12.06	(11.10–13.01)	14.33	(0.14–15.41)	
Physical activity					**<0.0001**
No	97.07	(96.53–97.60)	98.89	(0.99–99.22)	
Yes	2.93	(2.40–3,47)	1.11	(0.01–1.45)	

*N* = 24,117; <1.285 = 12,993; ≥1.285 = 11,124. *Chi-square test; CI%, Confidence interval; C-index cutoff point for assessing cardiovascular risk: ≥1.285 (female).Bold values indicate *p*-value < 0.05.

The binary logistic regression results indicated that, after adjustments, all variables remained significantly associated with cardiovascular risk according to C-Index, except for excessive alcohol consumption and smoking among men (Table 4).

**Table 4:** Association between sociodemographic variables and lifestyle habits with cardiovascular risk, estimated by binary logistic regression: National Health Survey, Brazil, 2013.

Variable	Male			Female		
	*p*-value*	OR	95% CI	*p*-value*	OR	95% CI
Intercept	<0.001	0.14	(0.10–0.19)	<0.001	0.12	(0.08–0.18)
Regions						
Southeast	-	Reference	-	-	Reference	-
Midwest	0.5817	0.95	(0.80–1.13)	**0.0031**	0.82	(0.72–0.93)
Northeast	0.4364	0.93	(0.78–1.12)	0.4954	1.05	(0.92–1.19)
North	0.8404	1.02	(0.83–1.25)	**0.0058**	0.81	(0.70–0.94)
South	0.4803	1.07	(0.88–1.31)	**<0.001**	0.77	(0.66–0.89)
Age range						
30–39	-	Reference	-	-	Reference	-
40–49	**<0.001**	1.99	(1.68–2.36)	**<0.001**	1.46	(1.28–1.65)
50–59	**<0.001**	4.32	(3.63–5.14)	**<0.001**	2.75	(2.36–3.20)
60–69	**<0.001**	6.61	(5.39–8.11)	**<0.001**	4.16	(3.52–4.92)
70–74	**<0.001**	7.77	(5.82–10.37)	**<0.001**	5.49	(4.22–7.14)
Race and ethnicity						
Yellow (Reference)	-	Reference	-	-	Reference	-
White	0.1149	0.55	(0.27–1.15)	**<0.001**	0.46	(0.31–0.70)
Indigenous	0.2640	0.67	(0.33–1.35)	0.5638	0.84	(0.46–1.53)
Brown	**<0.001**	0.79	(0.69–0.90)	0.6083	1.03	(0.92–1.15)
Black	**<0.001**	0.62	(0.49–0.78)	0.8801	1.01	(0.86–1.20)
Marital status						
Married	-	Reference	-	-	Reference	-
Divorced/Separated/Widowed	0.9532	0.99	(0.83–1.19)	0.1510	0.91	(0.80–1.03)
Single	0.2271	0.92	(0.80–1.05)	**0.0249**	0.88	(0.79–0.98)
Education						
No education/incomplete elementary school	**0.0439**	0.78	(0.61–0.99)	**<0.001**	1.72	(1.44–2.06)
Complete elementary education /incomplete high school	0.1049	0.80	(0.61–1.05)	**<0.001**	1.61	(1.32–1.97)
Complete high school / Incomplete higher education	0.8318	0.98	(0.78–1.22)	**<0.001**	1.41	(1.18–1.67)
Complete higher education/postgraduate degree	-	Reference	-	-	Reference	-
Paid occupation						
No	0.1085	1.14	(0.97–1.33)	**0.0206**	1.13	(1.02–1.26)
Yes	-	Reference	-	-	Reference	-
Income						
≤ 1 Minimum Wage	**0.0107**	0.72	(0.55–0.93)	**<0.001**	1.63	(1.31–2.02)
> 1 e ≤ 2 Minimum Wages	**0.0327**	0.76	(0.59–0.98)	**<0.001**	1.52	(1.23–1.88)
> 2 e < 5 Minimum Wages	0.1311	0.82	(0.64–1.06)	**0.0059**	1.35	(1.09–1.66)
≥ 5 Minimum Wages	-	Reference	-	-	Reference	-
Self-rated health						
Very good/Good	-	Reference	-	-	Reference	-
Fair	**<0.001**	1.39	(1.20–1.61)	**<0.001**	1.29	(1.17–1.43)
Poor/Very poor	**<0.001**	1.65	(1.30–2.08)	**<0.001**	1.61	(1.34–1.94)
Alcohol consumption						
No	.	.	.	-	Reference	-
Yes	.	.	.	0.0605	1.20	(0.99–1.46)
Smoker						
No	.	.	.	-	Reference	-
Yes	.	.	.	0.5565	1.04	(0.91–1.19)
Physical activity						
No	**<0.001**	1.82	(1.40–2.37)	**0.0074**	1.67	(1.15–2.44)
Yes	.	.	.	.	.	.

*N* = 42,693; Male *N* = 18,576; <1.344 = 13,290; ≥1.344 = 5,286. Female *N* = 24,117; <1.285 = 12,993; ≥1.285 = 11,124. *Binary logistic regression; OR, Odds ratio; CI%, Confidence interval. Models were adjusted for geographic region, age range, race and ethnicity, marital status, education, employment status, income, self-rated health status, and physical activity level.Bold values indicate *p*-value < 0.05.

In men, increasing age was associated with progressively higher odds of elevated cardiovascular risk according to the C-Index, being seven times higher among those aged 70–74 compared to those aged 30–39 (OR: 7.77; 95% CI: 5.82–10.37). Men identifying as Black or Brown had lower odds of cardiovascular risk compared to White men. Those with lower education levels had lower odds compared to those with higher education. Additionally, lower income was associated with higher odds of elevated cardiovascular risk. Poor self-rated health and physical inactivity doubled the odds of cardiovascular risk.

Among women, residing in the Central-West, North, and South regions was associated with lower odds of elevated cardiovascular risk compared to the Southeast. Increasing age was associated with progressively higher odds, with women aged 70–74 having five times higher odds than those aged 30–39 (OR: 5.49; 95% CI: 4.22–7.14). Women of Asian descent had lower odds, and single women had lower odds compared to married women. Low education, absence of paid work, and lower income were associated with higher odds of elevated cardiovascular risk. Additionally, poor health perception and physical inactivity significantly increased the likelihood of the outcome.

## 4 Discussion

This is the first study to assess cardiovascular risk in a representative sample of the Brazilian population using a C-Index cutoff point.

The present findings, which indicate that 39% of the Brazilian population is at elevated cardiovascular risk according to C-Index, are consistent with the 38.1% prevalence observed in a comparative study using the Global Risk Score of the Brazilian Society of Cardiology. However, these figures are higher than those obtained using the Framingham Heart Study indices (19.4%) and the European SCORE (14.6%), which reported lower prevalence rates (39). These variations may be attributed to different risk assessment methods, population characteristics, and the prevalence of risk factors such as hypertension and obesity, which are highly prevalent in Brazil (40–42).

Elevated systolic blood pressure and poor dietary habits are the leading risk factors for CVD mortality and disability-adjusted life years in Brazil (43). Only 3.4% of Brazilians meet the criteria for ideal cardiovascular health, indicating that the majority of the population is at high risk. In this context, the importance of using diverse tools to assess this condition in the Brazilian population becomes evident. The C-Index is thus a potentially useful tool in clinical practice in Brazil due to its cost-effectiveness and applicability (32).

The prevalence of cardiovascular risk according to the C-Index was higher among women than men. This result may be explained by differences in body fat distribution and metabolism. Although men tend to accumulate more visceral fat, women generally have a higher percentage of total body fat and, with aging and menopause, tend to redistribute this fat to the abdominal region (44–47). Since the C-Index uses waist circumference to assess cardiovascular risk, this redistribution in women may lead to a higher prevalence of risk compared to men.

The higher prevalence of cardiovascular risk in the state of Rio Grande do Norte and the regional differences observed in the regression analysis may be attributed to unequal access to food and health-related behaviors across different locations. A study on food acquisition in various regions of Brazil highlighted the relationship between dietary patterns and obesity rates. Specifically, a contrast in obesity levels was identified in Rio Grande do Norte compared to other states in the Northeast (48). Thus, food geography, socioeconomic conditions, and regional dietary habits have a significant impact on the cardiovascular health of the Brazilian population.

Age showed a strong and consistent association with cardiovascular risk related to central adiposity in both men and women, supporting previous studies indicating a rise in this risk among both men and women as they grow older (49, 50). Additionally, the C-Index, as an indicator of abdominal obesity, reflects fat accumulation in this region, which also tends to increase with aging (51, 52).

Regarding race and ethnicity, among men, individuals who identified as Black or Brown had a lower likelihood of cardiovascular risk compared to White individuals. Although this finding may seem counterintuitive, given that self-identified Black individuals often exhibit higher cardiovascular risk (53), it can be explained by the nature of the tool used, which also reflects abdominal fat accumulation. Studies have shown that, regardless of sex, Black individuals tend to have lower levels of visceral fat compared to White people, even when they have similar or higher total body fat percentages (54–56).

Among women, being single was associated with lower odds of cardiovascular risk compared to married women. In addition, women without paid employment were more likely to present cardiovascular risk. This finding can be partly explained by the burden of social and occupational demands (57, 58). Married women, especially those facing a double work shift or engaged in unpaid domestic labor, may be more exposed to stressors that increase cardiovascular risk (59).

The results of the analyses suggested opposite effects of income and education on cardiovascular risk between men and women. Among women, lower levels of education and income were associated with an increased risk, whereas among men, these same factors were linked to a lower risk. This discrepancy may reflect social inequalities and gender roles, which differently influence living conditions, access to healthcare, and health-related behaviors for men and women (60–63).

These findings are also consistent with studies linking education level and Body Mass Index (BMI), indicating that women with higher educational attainment tend to have lower BMI. For men, income plays a more significant role in increasing BMI, possibly due to different dietary patterns and greater access to ultra-processed foods among higher socioeconomic groups (64–66).

Poor or very poor self-rated health showed a significant association with the presence of cardiovascular risk. Studies have demonstrated that negative health perception is linked to higher cardiovascular mortality, both among individuals with a history of cardiovascular disease and those without prior conditions (67–69). Moreover, better self-rated health is significantly associated with lower risks of hypertension, hypercholesterolemia, smoking, obesity, and higher vegetable intake (70–74).

Physical activity showed a protective effect against cardiovascular risk related to central adiposity, aligning with well-established evidence emphasizing its role in preventing cardiovascular diseases (75, 76). Moderate to vigorous physical activity, as recommended by guidelines, has the potential to positively impact the reduction of cardiovascular mortality in the adult population (28). Additionally, physical activity is associated with reduced blood pressure, improved insulin sensitivity, and more favorable lipid profiles (77–79). The benefits of physical activity are observed across all ages, races, and sexes (80, 81).

The main limitation of this study lies in its cross-sectional design, which prevents the establishment of causal relationships between the variables. Additionally, the study was limited to individuals aged 30 to 74 years due to the cutoff point used, which excluded younger adults and older seniors, thus limiting the generalizability of the findings to these age groups. These constraints should be carefully considered when interpreting the implications of the results.

## 5 Conclusion

The prevalence of cardiovascular risk in the Brazilian population, as assessed by the Conicity Index, was high. Furthermore, significant associations were identified between cardiovascular risk and sociodemographic variables such as age, education, income, and region of residence, as well as lifestyle factors including physical activity and self-rated health. These findings highlight the importance of strategies aimed at preventing and controlling cardiovascular risk, taking into account both individual and social factors.

## Data Availability

The raw data supporting the conclusions of this article will be made available by the authors, without undue reservation.
